# Human Leucocyte Antigens as Prognostic Markers in Head and Neck Squamous Cell Carcinoma

**DOI:** 10.3390/cancers14153828

**Published:** 2022-08-07

**Authors:** Gerhard Dyckhoff, Christel Herold-Mende, Sabine Scherer, Peter K. Plinkert, Rolf Warta

**Affiliations:** 1 Department of Otorhinolaryngology, Head and Neck Surgery, University of Heidelberg, 69120 Heidelberg, Germany; 2 Division of Experimental Neurosurgery, Department of Neurosurgery, University of Heidelberg, 69120 Heidelberg, Germany; 3 Transplantation Immunology, Institute of Immunology, University of Heidelberg, 69120 Heidelberg, Germany

**Keywords:** HLA typing, genetic variation, HNSCC, survival, allele frequencies

## Abstract

**Simple Summary:**

Human leucocyte antigens (HLAs) play a crucial role in the immune defense by presenting antigens to T lymphocytes. The particular combination of HLA alleles determines a patient-specific presentation of cell-derived peptide fragments to the patrolling immune cells. Since deregulated protein expression and mutations are hallmarks of cancer, such abnormal peptides can be presented by tumor cells and eventually recognized, and the presenting tumor cells are then killed by T cells. Therefore, the HLA composition might influence the formation and fate of cancer and might differ between tumor patients and healthy individuals. We performed HLA typing of 84 patients with head and neck cancer, and the results were compared to survival. Two of fifty HLA alleles analyzed showed a significantly different frequency compared with the normal population (HLA-A*25 and HLA-C*06). Patients with HLA-C*04 had poorer oncological outcomes, while patients with HLA-A*02 had significantly better survival.

**Abstract:**

Background: The induction and regulation of immune responses depend on human leucocyte antigen (HLA) molecules that present peptides derived from mutated neoantigens or tumor-associated antigens to cytotoxic T cells. The natural variation of HLA molecules might differ between tumor patients and the normal population. Thus, there might be associations between the frequencies of HLA alleles and the survival of tumor patients. Methods: This issue was studied in a cohort of 84 patients with head and neck squamous cell carcinomas (HNSCCs) of different localizations. The cohort was followed up for more than 10 years. HLA-A/B/C CTS-PCR-SSP typing at 1 field level from blood samples was performed, and the results were correlated with survival. Results: HLA-A*02 was the most prevalent allele in our cohort and was present in 51.1% of patients. The HLA-A*25 and HLA-C*06 alleles exhibited a significantly higher frequency in cancer patients than in the normal population of 174 blood and kidney donors (*p* = 0.02 and *p* = 0.01, respectively, Fisher’s exact test). For HLA-C*04, a negative impact on overall survival in univariate analysis (*p* = 0.045) and a negative, but statistically insignificant effect on survival toward poorer survival in multivariate analysis (HR: 1.82; 95% CI: 0.99–3.34, *p* = 0.053) were observed. In addition, HLA-A*02 was also beneficial for overall survival and progression-free survival in multivariate analysis (HR 0.54; 95% CI: 0.31–0.92; *p* = 0.023). Conclusion: HLA-A*02 allele expression might not only predict better survival but might also indicate superior tumor antigen presentation and, thus, help to select patients who could benefit from T-cell-dependent immunotherapies.

## 1. Introduction

In addition to surgery, radiation, and chemotherapy, immunotherapy is currently an emerging fourth pillar of cancer therapy and has been intensively investigated in clinical trials. This approach includes the broadly used, clinically effective immune checkpoint inhibition to overcome cancer-associated T-cell exhaustion, adoptive T-cell transfer, and antitumor vaccination [1,2]. The major histocompatibility complex (MHC), which in humans is called the human leukocyte antigen (HLA), is of particular importance for these treatments since it determines the presentation of antigens to T cells and is the basis for the recognition and killing of cancer cells [3,4]. This presentation process starts by the degradation of all proteins eventually generated by a cell in the ubiquitin-proteasome pathway, followed by their transport into the endoplasmic reticulum. Peptides of eight to nine amino acids in length are bound to HLA class I molecules (HLA-A, HLA-B, and HLA-C) and presented as complexes on the cell surface to circulating cytotoxic T cells [5,6]. If recognized as nonself, mutated, or tumor-associated antigens by binding of the T-cell receptor, they are able to elicit tumor-specific T-cell immunity [7]. Since genetic instability and deregulation of gene expression are hallmarks of cancer and are especially common in head and neck squamous cell carcinomas (HNSCC), mutations and tumor-associated antigens are frequently found in these tumors [8,9]. Due to the inherited genetic variance of the HLA super locus, a variety of HLA molecules with distinct peptide presentation preferences can be generated by two alleles for HLA-A, -B, and C [10,11]. Therefore, the patient-specific composition of an HLA locus might already have implications for the onset of a macroscopic tumor, which would explain reports of altered HLA allele frequencies in tumor patients compared to healthy individuals [12]. However, even after tumor formation, the HLA composition might be relevant to the outcomes of cancer patients. It is known that the effectiveness of classical radio- and chemotherapies is often affected by the immune microenvironment of a tumor and thereby indirectly by the HLA composition of a patient [13,14,15]. Possible implications of the HLA traits of a patient for the success of immunotherapy have been increasingly studied. An interesting example is a recent analysis demonstrating that cancer patients with a homozygote HLA-A*03 haplotype had shorter survival after receiving immune checkpoint inhibitor therapy than heterozygous HLA-A*03 patients and patients without this allele [16]. In addition to these therapeutic implications, the HLA composition might serve as a prognostic marker, as has been described for HLA-A*11, HLA-B*13, HLA-B*35, and HLA-B*51 [12,17]. Therefore, the potential impact of HLA traits on the development of HNSCC and associations with relapse and survival in HNSCC might partly explain unresolved issues of unexpected outcome variance. To assess HLA composition and the potential impact of the HLA allele frequencies on PFS and OS, HLA-A/B/C CTS-PCR-SSP typing at 1 field level from blood samples of 84 HNSCC patients was performed.

## 2. Materials and Methods

### 2.1. Patients and Study Population

Blood samples from 84 patients (65 male and 19 female) with histologically diagnosed squamous cell carcinomas of the head and neck (HNSCCs) were obtained. The study was approved by the institutional ethics committee (S-70/99, amendment 09/01/2004), and written informed consent was obtained from each patient. The included patients were treated by curative surgery and risk-adopted adjuvant radiotherapy (aRT) or chemoradiotherapy (aCRT). Patients were followed from the date of first diagnosis to the end of the study, whereas patients who were still alive were censored. Progression-free survival (PFS) was calculated from registration date until the date of either death or relapse, thus censoring patients without any malignancy at the last follow-up. Relapse was considered local recurrence and lymph node or distant metastasis. Clinical data of the patients were assessed in an MS Access Database (Microsoft, Redmond, WA, USA) and obtained by reviewing the medical records, and follow-up data were obtained by telephone or written correspondence. The classification of the tumor was performed according to the TNM staging system of the International Union against Cancer (UICC). As a control population, we used a group of 174 Caucasian patients published by the University of Essen in the Allele Frequency Net Database (http://www.allelefrequencies.net/pop6001c.asp?pop_name=Germany%20Essen; accessed on 1 June 2022) composed of kidney and blood donors. HLA typing was performed by sequence-specific primer (SSP)-HLA typing.

### 2.2. DNA Isolation and HLA Typing

DNA was extracted from blood samples by a MasterPure DNA purification kit (Lucigen, Middleton, WI, USA) according to the manufacturer’s instructions. Molecular HLA typing results for HLA-A, HLA-B, and HLA C at 1 field level were obtained from the extracted DNA using HLA-A/B/C CTS-PCR-SSP typing Kits (CTS Sequence, Heidelberg, Germany) according to the manufacturer’s instructions (www.ctstransplant.org; accessed on 1 June 2022).

### 2.3. Statistics

Carrier frequency was computed as the percentage of patients with the respective HLA allele present. The statistical significance of frequency differences was measured by Fisher’s exact test. Survival time was measured from the end of the initial curative therapy. Correlations between the HLA subgroups and survival were assessed by Kaplan–Meier curves, the log-rank test, and univariate and multivariate Cox proportional hazard (PH) regression analyses. In the multivariate analysis, significant clinical covariates were integrated in addition to the HLA trait of interest. All computations were performed within the R statistical software environment (version 4.2.0) using the survival, survminer, forestmodel, ggpubr, and tidyverse packages [18,19]. *p* values less than 0.05 were considered significant.

## 3. Results

The study cohort consisted of 84 patients suffering from newly diagnosed HNSCC, 65 men (77.4%) and 19 women (22.6%). The average age of the patients was 63.5 +/− 10.5 years old, ranging from 42 to 91 years old. The median age was 63 years old. The median follow-up was 4.2 years (IQR 1.5–9.2 years). Tumor localization included the whole range of HNSCCs: oropharynx (ORO; n = 30; 35.7%), larynx (LAR; n = 17; 20.2%), hypopharynx (HYPO; n = 12; 14.3%), oral cavity (n = 11; 13.1%), carcinoma of unknown primary origin (CUP; n = 7; 8.3%), and nasal cavity and paranasal sinuses (NC/PNS; n = 7; 8.3%). More than 60% of the patients suffered from advanced UICC stage IV tumors (Table 1).

**Figure 1:**
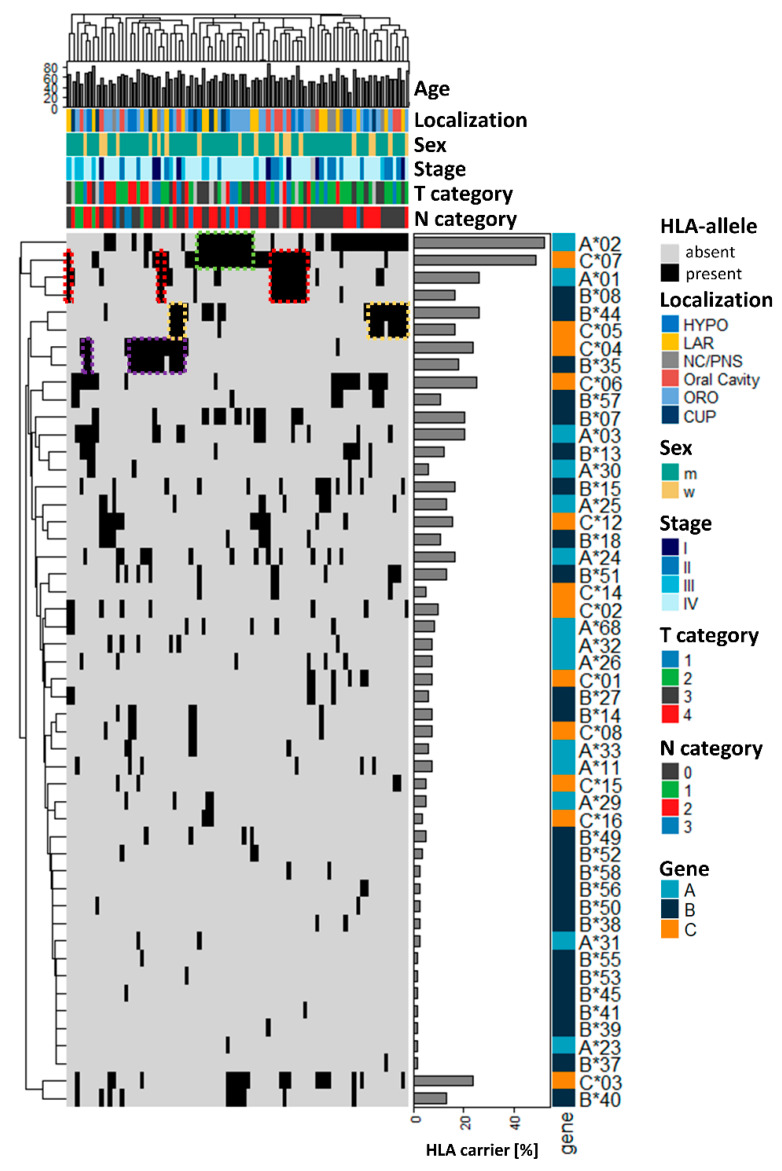
Survey of the main clinical characteristics of the cohort of 84 HNSCC patients in correlation with HLA allele frequencies. Ordering of the patients based on hierarchical clustering of HLA composition did not reveal enrichment of clinical parameters. HLA—human leukocyte antigen; HYPO—hypopharynx; LAR—larynx; NC/PNS—nasal cavity/paranasal sinuses; ORO—oropharynx; CUP—carcinoma of unknown primary, age at diagnosis in years; T—tumor; N—lymph node.

In this study sample, analysis of HLA class I alleles revealed 50 distinguishable HLA alleles, which are visualized together with the clinicopathological information of the patients in Figure 1. HLA-A*02 (52.4%) and HLA-C*07 (48.8%) were the most common alleles, and at least one of them was present in more than 75% of HNSCC patients. Ordering of the patients (columns) by hierarchical clustering based on their HLA composition did not reveal an obvious association between the clinical parameters and specific HLA alleles (Figure 1).

**Figure 2:**
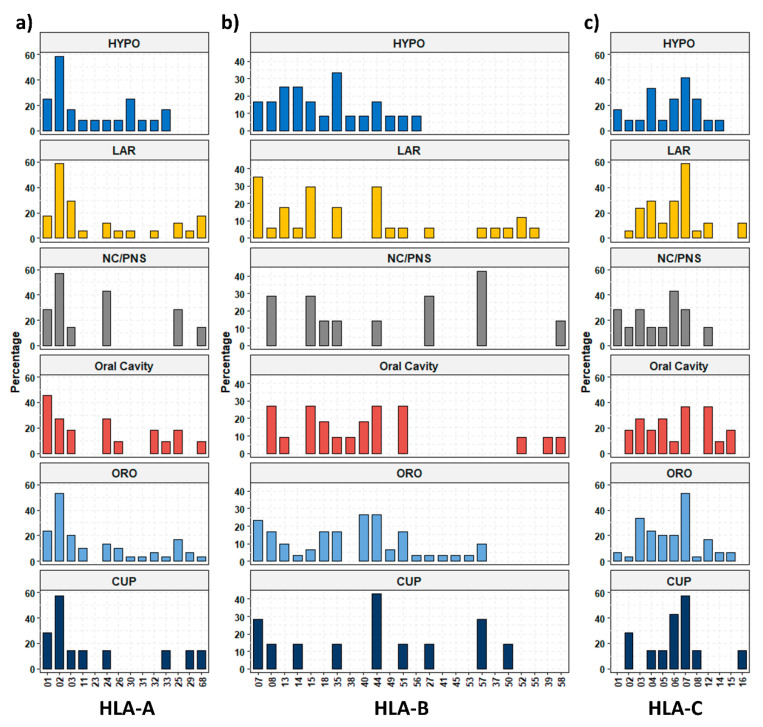
Frequencies of human leucocyte antigen (HLA) class I alleles dependent on different tumor localizations. (**a**) HLA-A alleles, (**b**) HLA-B alleles, and (**c**) HLA-C alleles. HYPO—hypopharynx; LAR—larynx; NC/PNS—nasal cavity/paranasal sinuses; ORO—oropharynx; CUP—carcinoma of unknown primary.

However, additional ordering of the HLA alleles (rows) visualized several blocks of two-allele haplotypes of more common alleles, such as A*02/C*07 (green) in 22.6%, B*35/C*04 in 17.9% (purple), and B*44/C*07 in 16.6% (yellow), or even triple allele haplotypes A*01/B*08/C*07 in 15.5% of the patients. This outcome might suggest an association of these alleles in a subgroup of the patients. To investigate whether the composition of the HLA alleles differed in HNSCC patients compared with healthy individuals, we analyzed the percentage of patients carrying a specific allele in our study sample and compared this finding to a dataset of German blood and kidney donors derived from the Allele Frequency Net Database [20] (Table 2, Table 3 and Table 4). Despite the limited size of the two cohorts, we identified two alleles exhibiting a significantly different incidence in the normal population. There was a 2.8-fold increased percentage for HLA-A*25 (normal 8/172, tumor 11/84, *p* = 0.02) and a 2.1-fold increase in HLA-C*06 (normal 21/172, tumor 21/84, *p* = 0.01) in HNSCC patients. For all other HLA alleles assessed, there was no significant difference identified. Furthermore, the HLA carrier frequency depending on the tumor location is shown in Figure 2 (Table S1). For the most prevalent HLA-A*02 allele, we found a very high frequency of almost 60% in most of the tumor locations, except for oral cavity carcinoma (27.2%) (Figure 2a). For HLA-C*07, the second most common allele, we found more heterogeneous expression, ranging from 28.6% in NC/PNS to 58.8% in LAR (Figure 2c). For the other HLA alleles, especially of the HLA-B type, a quite heterogeneous presence was observed but could not be attributed to a certain tumor localization (Figure 2a–c).

**Figure 3:**
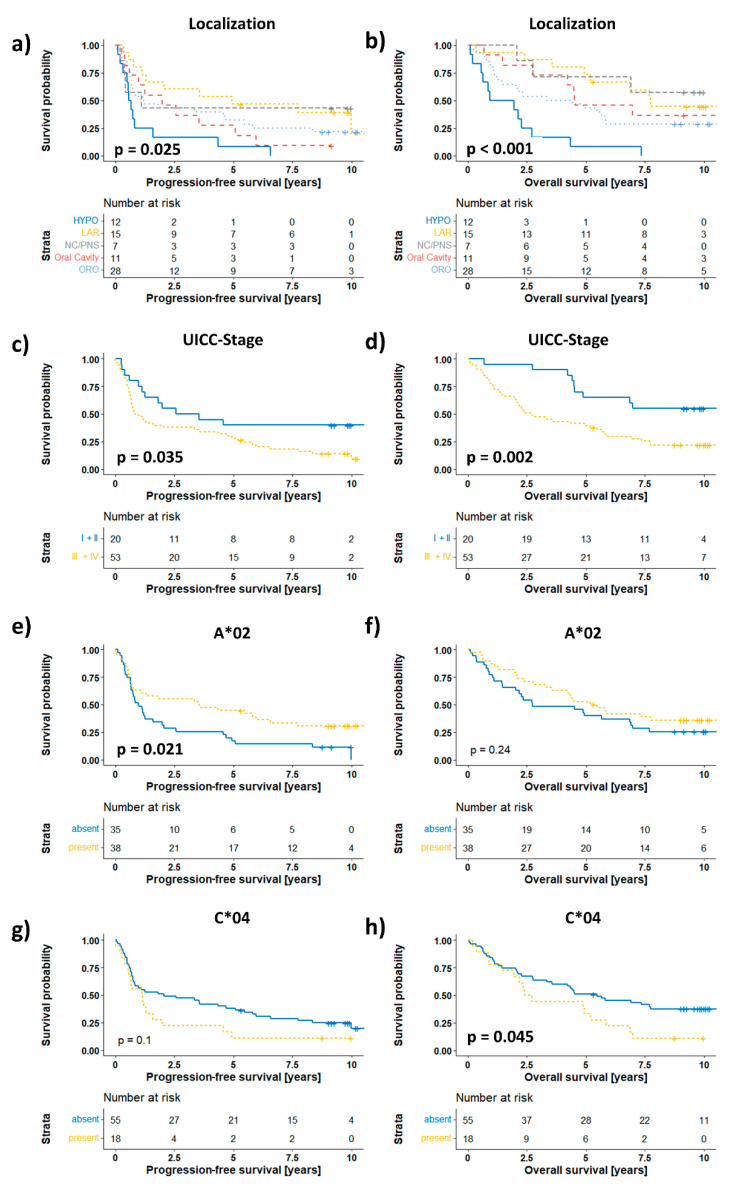
Associations of clinical covariates and HLA-A*02 and HLA-C*04 with progression-free and overall survival. Kaplan–Meier curves of (**a**,**b**) localization, (**c**,**d**) UICC stage, (**e**,**f**) HLA-A*02, and (**g**,**h**) HLA-C*04 visualize nonparametric survival analysis. HYPO—hypopharynx; LAR—larynx; NC/PNS—nasal cavity/paranasal sinuses; ORO—oropharynx; *p* values in the graphs depict the univariate/multivariate Cox PH model results.

Finally, we assessed the associations of HLA prevalence with progression-free survival (PFS) and overall survival (OS). Here, we analyzed our dataset in a two-stage process to account for the heterogeneous composition of the investigated cohort. In the first step, we tested clinical covariates such as sex, age, and UICC stage in a univariate log-rank test and treatment (surgery ± a(C)RT) and localization by a Cox PH model for their associations with patient outcomes, and we visualized the results with Kaplan–Meier curves (Figure 3, Figures S1 and S2; Tables S2 and S3). Here, we found significantly different PFS (0.025) and OS (*p* < 0.001) of patients depending on their tumor localization (Figure 3a–b). In addition, a higher UICC stage was associated with shorter PFS (*p* = 0.035) and OS (*p* = 0.002) (Figure 3c–d). Age, treatment, and sex had no significant associations with PFS or OS (Figures S1 and S2). Next, we tested every individual allele that was present in at least 5 patients for its associations with OS and PFS (Tables S2 and S3). Interestingly, the presence of the most prevalent HLA-A*02 allele was significantly associated with a longer PFS (*p* = 0.021, Figure 3e) but not OS (*p* = 0.24, Figure 3f).

**Figure 4:**
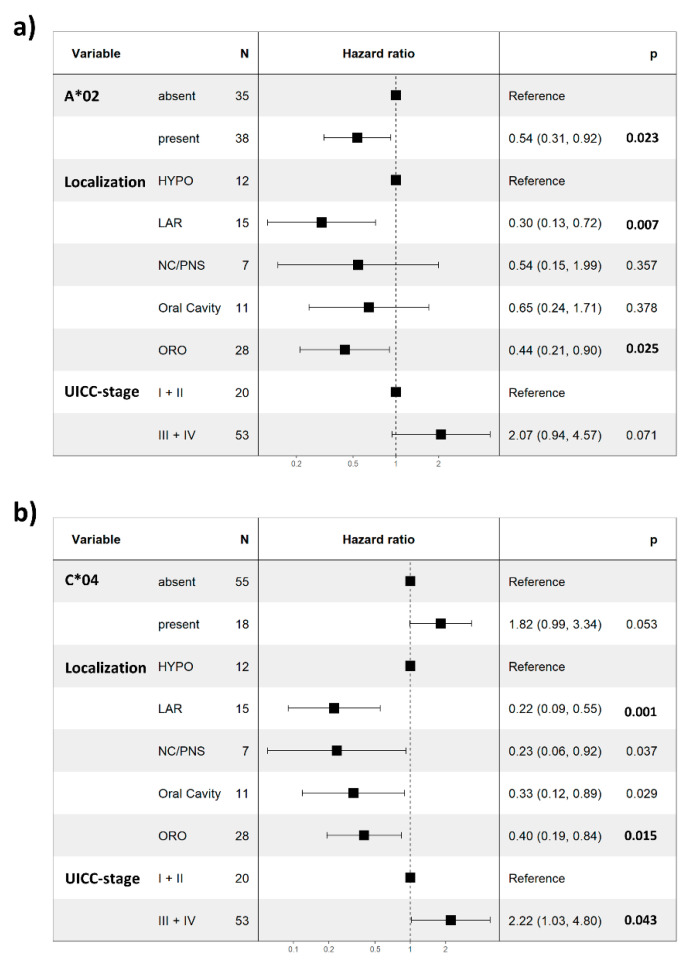
Multivariate Cox PH model of progression-free and overall survival. Forest plot visualizes the results of a multivariate Cox PH model including localization and UICC stage of (**a**) HLA-A*02 (PFS) and (**b**) HLA-C*04 (OS). Values depict hazard ratios (lower and upper 95% confidence intervals). HYPO—hypopharynx; LAR—larynx; NC/PNS—nasal cavity/paranasal sinuses; ORO—oropharynx; *p* values in the graphs depict the multivariate Cox PH model results.

Interestingly, for the presence of HLA-C*04, we observed an opposite trend of a shorter PFS (*p* = 0.1, Figure 3g), while the association was significant for OS (*p* = 0.045, Figure 3h). Furthermore, correction for the influences of stage and localization on PFS by a multivariate Cox PH model revealed HLA-A*02 as a statistically significant independent prognostic factor of PFS in our HNSCC cohort (HR 0.54; 95% CI: 0.31–0.92; *p* = 0.023; Figure 4a and Table S4). However, when correcting for localization and stage in a multivariate Cox PH model of overall survival, HLA-C*04 failed to reach the level of significance as an independent prognostic factor (*p* = 0.053; HR: 1.82; 95% CI: 0.99–3.34; Figure 4b and Table S5). Although correction of the association of HLA-A*02 in this multivariate model substantially improved the prognostic power for overall survival, it still did not reach the level of significance (*p* = 0.063; HR: 0.59; 95% CI: 0.34–1.03; Figure S1e and Table S5). Finally, in order to avoid this possible bias by HPV-induced oropharyngeal cancers we have conducted a survival analysis excluding the patients with oropharyngeal cancer (Figures S3–S5). Despite the decreased patient number of 45, all major findings are still valid and supported by the results. This suggests that our findings are very robust and that our cohort is very homogenous.

## 4. Discussion

In the present study, we investigated the prevalence of HLA class I alleles in a cohort suffering from HNSCCs, including various localizations and UICC stages. Our results allow for an overview of the distribution of the common HLA alleles in HNSCC patients, uncovering a higher frequency of HLA-A*25 and HLA-C*06 in HNSCC than in a healthy control group and suggesting an association of the HLA-C*04 and HLA-A*02 genotypes with the OS and PFS of patients. Interestingly, these survival associations had opposing directions, namely, a shorter OS in the presence of C*04 and a longer PFS time in the presence of A*02, even after correction for the clinical covariates of localization and UICC stage.

The observed association of the HLA composition with the outcomes of HNSCC patients (PFS and OS) is in line with previously conducted studies, also suggesting an influence of HLA allele traits on survival [12,17,21,22]. For example, Wichmann et al. investigated a comparable HNSCC cohort (n = 90) and reported HLA-B*13, HLA-B*35, and HLA-B*51 as independent predictors of PFS in HNSCCs [12,22]. However, in our cohort, there were no significant associations of these HLA-B alleles with either PFS or OS. Another study conducted by Tisch et al. investigated a larger HNSCC cohort (n = 141) and found significantly shorter OS of HNSCC patients with the HLA-A*11 antigen [17]. Again, we could not reproduce this finding in our cohort. The reason for this inability might be that A*11 is a relatively rare HLA antigen (~10% in Tisch et al., ~5% Wichmann et al. and in our cohort) and the 84 patients in our cohort might not have been sufficient to achieve a statistically robust result for A*11. However, Tisch et al. also described longer survival of HLA-A*02-positive patients, albeit without statistical significance (*p* = 0.15). This trend supports our finding of a similarly strong trend for OS in our cohort (*p* = 0.06). In addition, we were able to demonstrate a statistically significant, independent association of HLA-A*02 with longer PFS in a multivariate setting, which was not analyzed in the manuscript by Tisch et al. In line with this observation, an analysis of OS in the HNSCC with the Cancer Genome Atlas cohort backed the strong trend for longer survival of HLA-A*02-positive patients (*p* = 0.146), but it also did not analyze PFS [21]. Moreover, we observed a statistically significant and, in a multivariate setting, independently shorter OS for patients with the HLA-C*04 genotype. This association with the survival of HNSCC patients seems to have not been previously reported. In summary, we described a survival association with HLA traits that is partially supported by other reports and deserves further investigation to decipher the biological consequences of the observed survival differences in greater depth.

A widely used theory to explain survival differences resulting from distinct HLA genotypes is based on genotype-specific peptide binding affinities. Ultimately, this affinity results in differential peptide presentation by MHC molecules on the cell surface if their sequence matches the preferred amino acids at the peptide binding positions [21,23]. The presentation of neo- and tumor-associated antigens might then result in better recognition by T cells and higher cytotoxic activity, which have both been associated with improved patient survival [24,25,26]. For instance, reports of HLA-A*02-specific CD8+ cytotoxic T cells recognizing the oncogenes EGFR and p53 in the circulation of HNSCC patients could account for the observed improved outcomes of HNSCC patients with this genotype [27,28]. However, these outcomes cannot explain the observed associations of HLA-C*04 or HLA-A*11 with poorer patient outcomes and, therefore, require further investigation.

In addition, the observed significant overrepresentation of HLA-A*25 and HLA-C*06 when comparing the frequencies of the HLA alleles in HNSCC patients and a healthy control group also warrants future validation in additional well-defined study samples. Apart from this finding, we did not observe any other significant differences, suggesting only a weak association of the HLA genotypes with the incidence of classical HNSCC. This finding is in line with Wichmann et al., who found HLA antigen frequencies almost equal to the general population in Germany. However, they further observed deviations only on the HLA-B locus: the frequencies of HLA-B*44 were lower, and those of HLA-B*13, HLA-B*52, and HLA-B*57 were increased (*p* < 0.05) [12]. Similarly, in a Finnish HNSCC study group, Koskinen et al. found only insignificant differences from the controls. However, these authors only investigated HLA class II antigens [29]. Reinders et al. described no differences in the HLA-A and HLA-C loci in a Dutch HNSCC cohort. Statistically significant differences were only observed for HLA-B*40 between the normal population and the subgroup of patients with a tumor of the oral cavity and for B*35 with the metastasized patient group [30]. Overall, between HNSCC tumor patients and control populations, there seem to be only slight differences in the frequency distributions of the HLA class I system.

Notably, a limitation of our study might that the group size of our study cohort, although substantial, might have been too small for statistically robust variance analysis; therefore, the comparison with the normal population should be regarded with caution. Furthermore, low-resolution HLA typing allowed only for superficial allocation into separate HLA groups. Moreover, only MHC class I genes were analyzed, whereas MHC class II genes might influence the outcome of the patients in a similar way. Finally, another potential drawback is that, for several oropharyngeal cancers in our study group, p16 status was missing so that HPV as an important prognostic marker could not be considered.

## 5. Conclusions

In our study cohort consisting of 84 HNSCC patients, significant differences were restricted to two HLA class I alleles (HLA-A*25 and HLA-C*06). Thus, there seem to be only slight differences in the frequency distribution between tumor patients and healthy controls. Nevertheless, for HLA-C*04, we identified a significant, negative correlation with OS, while HLA-A*02 was correlated with longer OS and, in a multivariate setting, it was significantly associated with longer PFS of the patients. For the most prevalent HLA-A*02 allele, a positive correlation has been found in other studies. Since it has been associated with the presentation of EGFR and p53 peptides by others [27,28], HLA-A*02 might be a promising candidate for the selection of patients who could benefit from immunotherapeutic interventions. Especially in times of broadly available highly capable and affordable next-generation sequencing, this type of data could be routinely assessed and integrated into patient consulting and therapy concepts.

## Figures and Tables

**Table 1:** Study sample description.

Variable	Category	n	%
Age (years)	<50	15	17.9
	50–59	30	39.3
	60–69	27	28.6
	70–79	9	10.7
	>80	3	3.6
Sex	Male	65	77.4
	Female	19	22.6
Localization	Hypopharynx	12	14.3
	Larynx	17	20.2
	Oropharynx	30	35.7
	Oral cavity	11	13.1
	Nasal cavity/paranasal sinuses	7	8.3
	CUP	7	8.3
T category	T1	13	15.5
	T2	28	33.3
	T3	17	20.2
	T4	17	20.2
	Unknown/n.a. (CUP)	9	10.7
N category	N0	36	43.4
	N1	5	6.0
	N2	35	42.2
	N3	7	8.4
	Unknown	1	1.2
UICC stage	I	8	9.6
	II	14	16.9
	III	11	13.3
	IV	50	60.2
	Unknown	1	1.2
Therapy	Surgery only	40	47.6
	Surgery + aCRT	17	20.2
	Surgery + aRT	27	32.1

CUP—carcinoma of unknown primary; n.a.—not appropriate; n—number; T—tumor; N—lymph node; aCRT—adjuvant chemoradiotherapy; aRT—adjuvant radiotherapy.

**Table 2:** Fourteen different HLA-A alleles were present in HNSCC patients; one exhibited a significantly different frequency compared to the normal population (gray).

HLA-Type	Normal (% (n))	Tumor (% (n))	*p* Value
HLA-A			
HLA-A*01	25.9 (45)	26.2 (22)	1.00
HLA-A*02	51.1 (89)	52.4 (44)	0.89
HLA-A*03	28.7 (50)	20.2 (17)	0.17
HLA-A*11	8.6 (15)	7.1 (6)	0.81
HLA-A*23	3.4 (6)	1.2 (1)	0.43
HLA-A*24	19.0 (33)	16.7 (14)	0.73
HLA-A*25	4.6 (8)	13.1 (11)	0.02
HLA-A*26	6.3 (11)	7.1 (6)	0.79
HLA-A*29	4.0 (7)	4.8 (4)	0.75
HLA-A*30	4.6 (8)	6.0 (5)	0.76
HLA-A*31	6.9 (12)	2.4 (2)	0.16
HLA-A*32	3.4 (6)	7.1 (6)	0.21
HLA-A*33	3.4 (6)	6.0 (5)	0.34
HLA-A*68	12.1 (21)	8.3 (7)	0.40

Gray is sufficient to highlight the significantly different HLA-type.

**Table 3:** Thirty-one different HLA-B alleles were present in HNSCC patients; none exhibited a significantly different frequency compared to the normal population.

HLA-Type	Normal (% (n))	Tumor (% (n))	*p* Value
HLA-B			
HLA-B*07	25.3 (44)	20.2 (17)	0.44
HLA-B*08	25.3 (44)	16.7 (14)	0.15
HLA-B*13	5.2 (9)	11.9 (10)	0.07
HLA-B*14	3.4 (6)	7.1 (6)	0.21
HLA-B*15	14.4 (25)	16.7 (14)	0.71
HLA-B*18	4.6 (8)	10.7 (9)	0.10
HLA-B*27	13.2 (23)	6.0 (5)	0.09
HLA-B*35	13.2 (23)	17.9 (15)	0.35
HLA-B*37	1.7 (3)	1.2 (1)	1.00
HLA-B*38	4.6 (8)	2.4 (2)	0.51
HLA-B*39	5.7 (10)	1.2 (1)	0.11
HLA-B*40	17.2 (30)	13.1 (11)	0.47
HLA-B*41	3.4 (6)	1.2 (1)	0.43
HLA-B*42	0.0 (0)	0.0 (0)	1.00
HLA-B*44	17.2 (30)	26.2 (22)	0.10
HLA-B*45	0.6 (1)	1.2 (1)	0.55
HLA-B*46	0.0 (0)	0.0 (0)	1.00
HLA-B*47	0.0 (0)	0.0 (0)	1.00
HLA-B*48	0.6 (1)	0.0 (0)	1.00
HLA-B*49	2.3 (4)	4.8 (4)	0.28
HLA-B*50	1.7 (3)	2.4 (2)	0.66
HLA-B*51	10.3 (18)	13.1 (11)	0.53
HLA-B*52	1.1 (2)	3.6 (3)	0.33
HLA-B*53	0.0 (0)	1.2 (1)	0.33
HLA-B*54	0.6 (1)	0.0 (0)	1.00
HLA-B*55	6.3 (11)	1.2 (1)	0.11
HLA-B*56	2.3 (4)	2.4 (2)	1.00
HLA-B*57	5.2 (9)	10.7 (9)	0.12
HLA-B*58	2.9 (5)	2.4 (2)	1.00
HLA-B*59	0.6 (1)	0.0 (0)	1.00
HLA-B*73	0.0 (0)	0.0 (0)	1.00

**Table 4:** Thirteen different HLA-C alleles were present in HNSCC patients; one exhibited a significantly different frequency compared to the normal population (gray).

HLA-Type	Normal (% (n))	Tumor (% (n))	*p* Value
HLA-C			
HLA-C*01	10.9 (19)	7.1 (6)	0.38
HLA-C*02	5.2 (9)	9.5 (8)	0.19
HLA-C*03	30.5 (53)	23.8 (20)	0.30
HLA-C*04	17.2 (30)	23.8 (20)	0.24
HLA-C*05	12.1 (21)	16.7 (14)	0.34
HLA-C*06	12.1 (21)	25 (21)	0.01
HLA-C*07	59.2 (103)	48.8 (41)	0.14
HLA-C*08	4.0 (7)	7.1 (6)	0.36
HLA-C*12	9.8 (17)	15.5 (13)	0.21
HLA-C*14	1.7 (3)	4.8 (4)	0.22
HLA-C*15	4.0 (7)	4.8 (4)	0.75
HLA-C*16	1.1 (2)	3.6 (3)	0.33
HLA-C*17	1.7 (3)	0.0 (0)	0.55

Gray is sufficient to highlight the significantly different HLA-type.

## Data Availability

The data presented in this study are available on request from the corresponding author.

## Supplementary Materials

The following supporting information can be downloaded at: https://www.mdpi.com/article/10.3390/cancers14153828/s1, Figure S1: Overall survival; Figure S2: Progression-free survival; Figure S3: Univariate survival analysis for PFS and OS excluding patients with oropharyngeal cancer. Localization, UICC stage, HLA-A*02, HLA-C*04 are visualized by Kaplan–Meier curves. Figure S4: Multivariate survival analysis for PFS excluding patients with oropharyngeal cancer. Figure S5: Multivariate survival analysis for OS excluding patients with oropharyngeal cancer. Table S1: frequency and count of HLA-alleles; Table S1: frequency and count of HLA-alleles; Table S2: Progression-free survival univariate results; Table S3: Overall survival univariate results; Table S4: Progression-free survival multivariate results; Table S5: Overall survival multivariate results
