# Supplementary material for: Human Leucocyte Antigens as Prognostic Markers in Head and Neck Squamous Cell Carcinoma
Source: Cancers (Basel). 2022 Aug 7;14(15):3828. doi: 10.3390/cancers14153828 (PMC9367389; doi:10.3390/cancers14153828)
Supplement: Supplementary file 1 [file cancers-14-03828-s001.zip › Figure S1-S5.pdf]

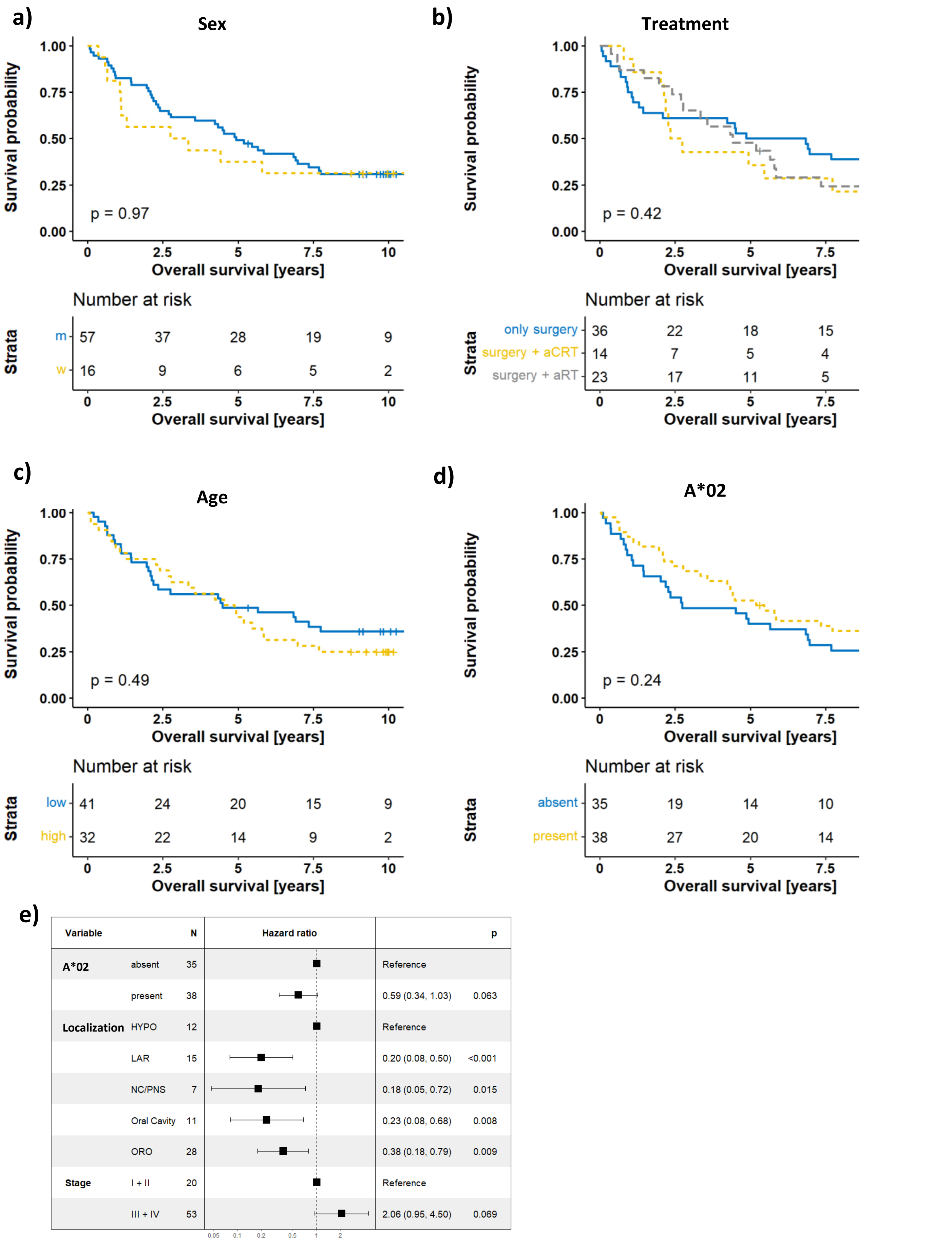

Figure S1. Associations of clinical covariates and HLA-A*02 overall survival. Kaplan-Meier curves of **(a)** sex, **(b)** treatment, **(c)** age and **(d)** HLA-A*02 visualize nonparametric survival analysis. **(e)** HLA-A*02 multivariate Cox PH model survival analysis corrected for localization and UICC stage. HYPO: hypopharynx; LAR: larynx; NC/PNS: nasal cavity/paranasal sinuses; ORO: oropharynx; p values in the graphs depict the univariate/multivariate Cox PH model results.

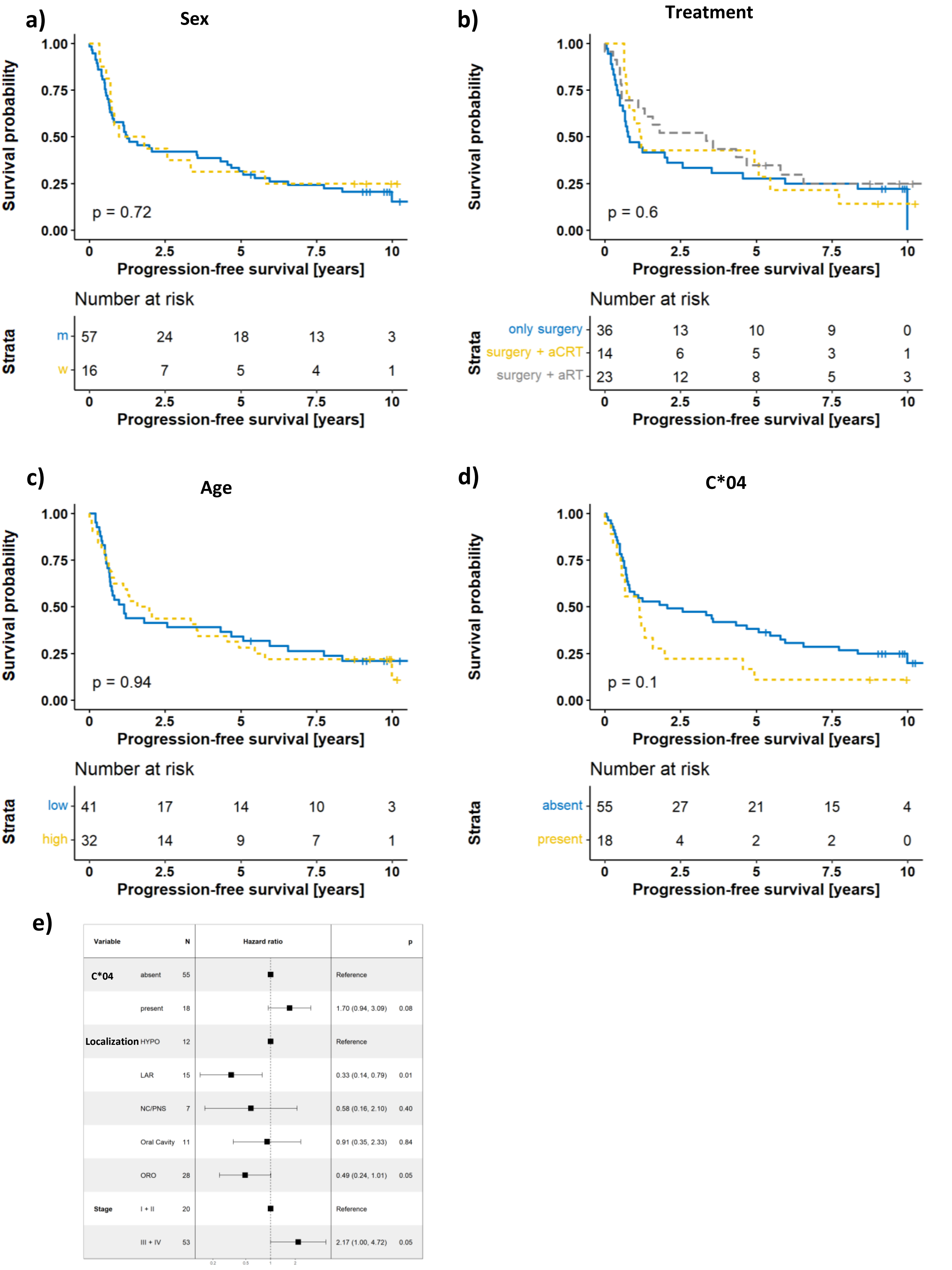

Figure S2. Associations of clinical covariates and HLA-C*04 Progression-free survival. Kaplan-Meier curves of **(a)** sex, **(b)** treatment, **(c)** age and **(d)** HLA-A*02 visualize nonparametric survival analysis. **(e)** HLA-C*04 multivariate Cox PH model survival analysis corrected for localization and UICC stage. HYPO: hypopharynx; LAR: larynx; NC/PNS: nasal cavity/paranasal sinuses; ORO: oropharynx; p values in the graphs depict the univariate/multivariate Cox PH model results.

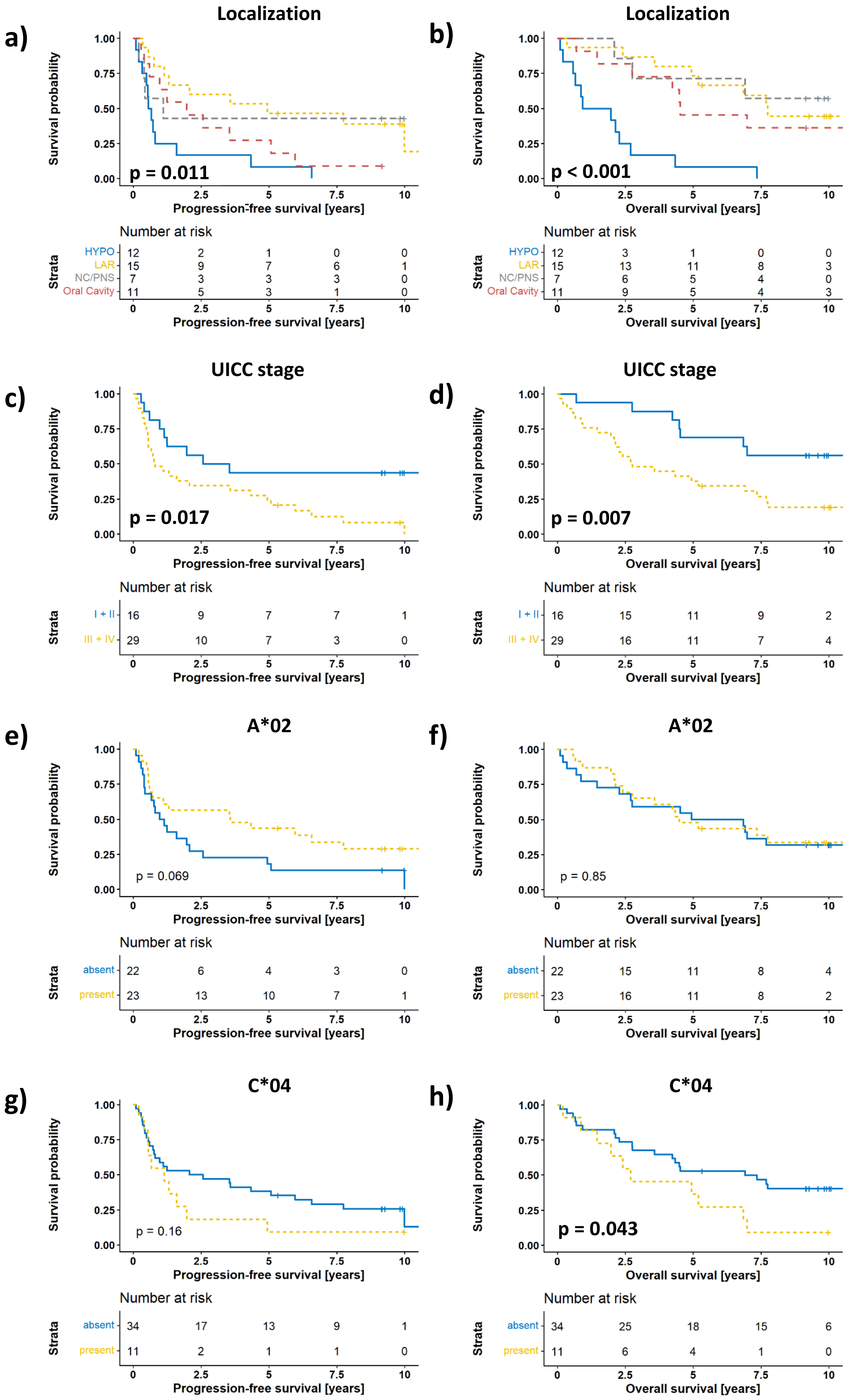

Figure S3. Associations of clinical covariates and HLA-A*02 and HLA-C*04 with progression-free and overall survival excluding the patients with oropharyngeal cancer. Kaplan-Meier curves of **(a-b)** localization, **(c-d)** UICC stage, **(e-f)** HLA-A*02 and **(g-h)** HLA-C*04 visualize nonparametric survival analysis. HYPO: hypopharynx; LAR: larynx; NC/PNS: nasal cavity/paranasal sinuses; p values in the graphs depict the univariate/multivariate Cox PH model results.

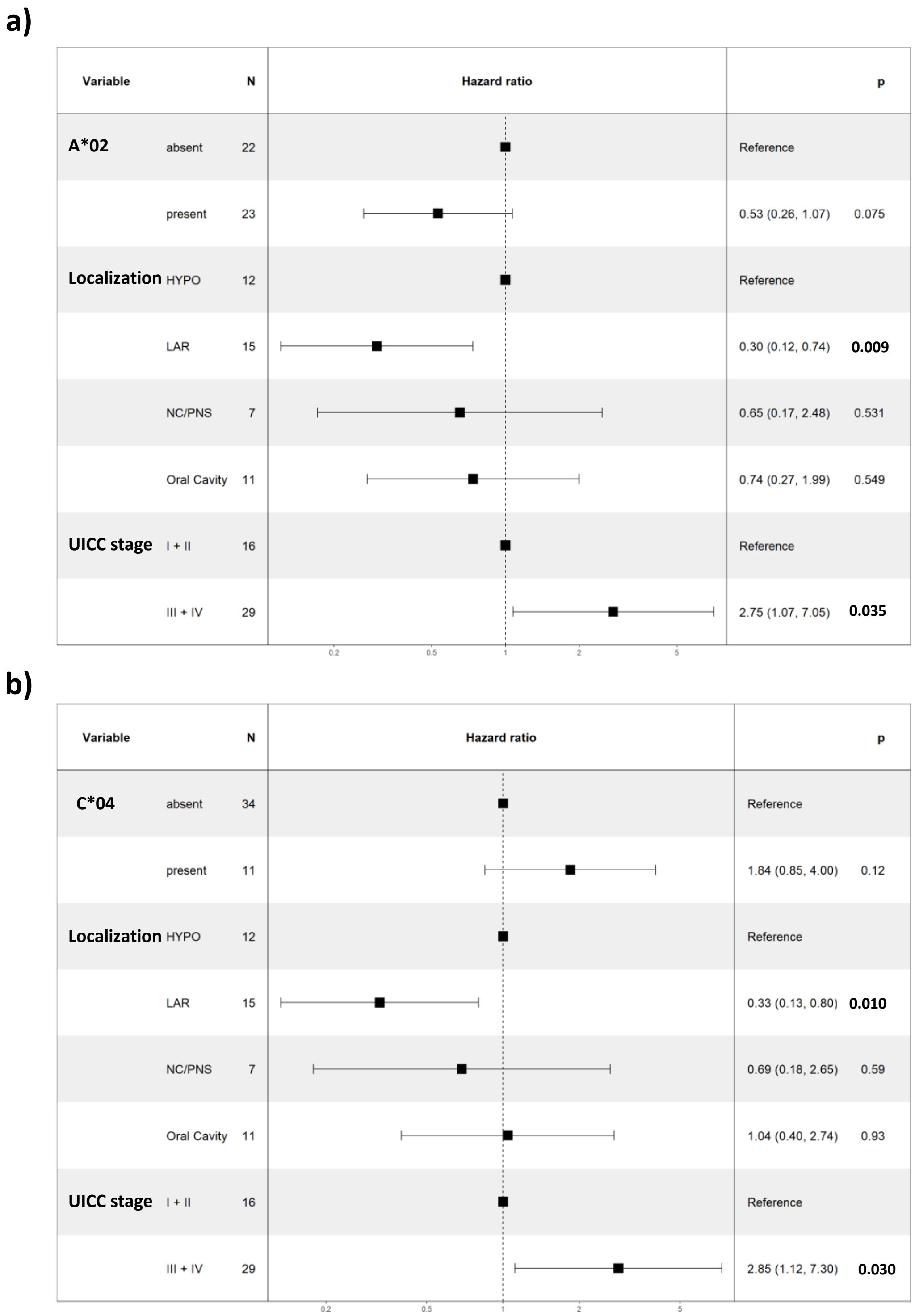

Figure S4. Multivariate Cox PH model of progression-free survival excluding the patients with oropharyngeal cancer. Forest plot visualizes the results of a multivariate Cox PH model including localization, UICC stage **(a)** HLA-A*02 and **(b)** HLA-C*04. Values depict hazard ratios (lower and upper 95% confidence intervals). HYPO: hypopharynx; LAR: larynx; NC/PNS: nasal cavity/paranasal sinuses; p values in the graphs depict the multivariate Cox PH model results.

a)

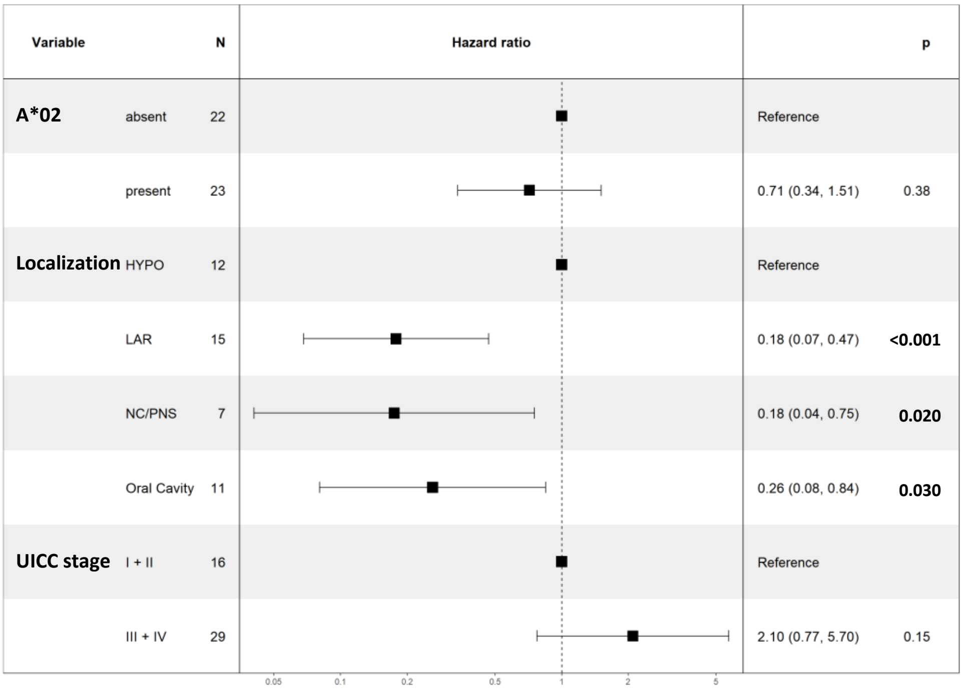

b)

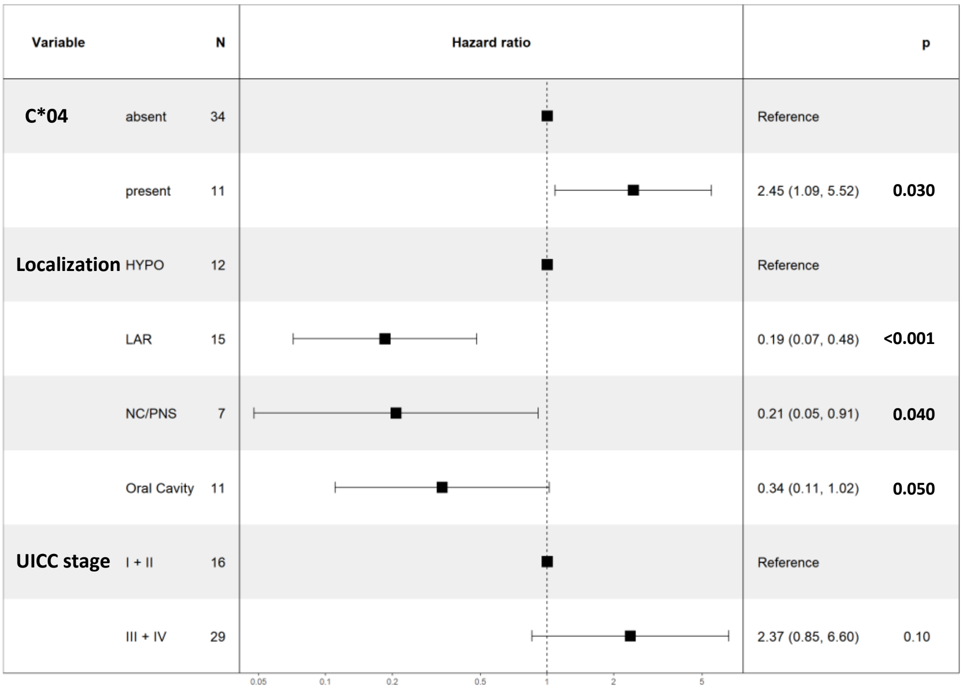

Figure S5. Multivariate Cox PH model of overall survival excluding the patients with oropharyngeal cancer. Forest plot visualizes the results of a multivariate Cox PH model including localization, UICC stage **(a)** HLA-A*02 and **(b)** HLA-C*04. Values depict hazard ratios (lower and upper 95% confidence intervals). HYPO: hypopharynx; LAR: larynx; NC/PNS: nasal cavity/paranasal sinuses; p values in the graphs depict the multivariate Cox PH model results.
